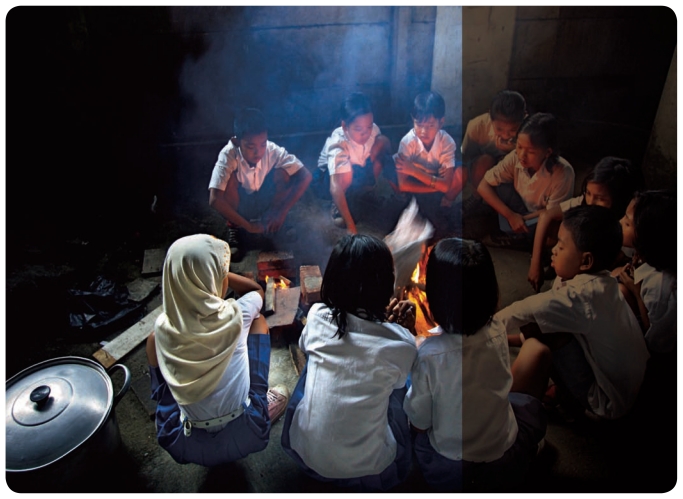# INTERNATIONAL ENVIRONMENTAL HEALTH: Assessing the Global Composite Impact of Chemicals on Health

**DOI:** 10.1289/ehp.119-a162

**Published:** 2011-04

**Authors:** Bob Weinhold

**Affiliations:** **Bob Weinhold**, MA, has covered environmental health issues for numerous outlets since 1996. He is a member of the Society of Environmental Journalists

The total role that the environment plays in contributing to premature death and disability remains sketchy, but researchers working with the World Health Organization (WHO) continue to flesh out the details. Now four WHO researchers have estimated the global burden of certain toxic substances for which adequate data are available.^1^ They have calculated that in 2004^2^ at least 8.3% of all preventable deaths and 5.7% of the preventable portion of the metric known as disability-adjusted life years (DALYs, which address a blend of death and disease impacts) were caused by the toxics analyzed.^1^ Their estimates include health end points such as cardiovascular disease, cancers, neuropsychiatric disorders, asthma, chronic obstructive pulmonary disease, respiratory infections, and birth defects.^3^

The leading contributors to premature death and DALYs were indoor solid fuel combustion (1,965,000 deaths and 41,009,000 DALYs), outdoor air pollution (1,212,000 deaths and 8,747,000 DALYs), secondhand tobacco smoke (SHS; 603,000 deaths and 10,913,000 DALYs), chronic occupational exposures (581,000 deaths and 6,763,000 DALYs), chemicals involved in unintentional, nonoccupational acute poisonings (210,000 deaths and 4,603,000 DALYs), and suicide attempts using pesticides (186,000 deaths and 4,420,000 DALYs). Other toxics included in the global totals were asbestos, lead, arsenic in drinking water (but only in Bangladesh), and a few other chemicals, including some typically encountered in occupational settings.^1^

Children under age 15 years were a highly susceptible group, suffering 54% of the total DALY burden. That includes 80% of the burden imposed by lead, 75% of that of indoor solid fuel use, 61% of that of SHS, 19% of that of acute accidental poisonings, and 10% of that of outdoor air pollution.^1^

The estimates include only a small fraction of all plausible chemical actors, says lead author Annette Prüss-Ustün. Among the thousands of toxics and pathways not included were mercury, dioxins, cadmium, radioactive substances, chronic pesticide exposures, active tobacco smoking, nonurban outdoor air pollutants, site-specific pollution hot spots, and chemicals whose actions are altered by climate change.

Nevertheless, Prüss-Ustün and colleagues think their analysis covers a high percentage of all acute impacts and a moderately high portion of all elevated chronic occupational exposures, although only a modest fraction of all chronic exposures in the general population, and almost none of the impacts such as developmental damage, degradation of specific organs or body systems, or harm caused by the extensive synthetic chemical body burden documented to occur in many people.^1^

The WHO team’s findings were based on evidence provided in numerous existing studies, including meta-analyses when available. To estimate the burdens, the team usually used a comparative risk assessment method that attempts to identify harm caused by factors such as the concentration of a substance above safe levels, such as particulate matter (PM) emitted by vehicles or combustion of solid fuels. When there was substantial but limited data that precluded using this method alone, expert opinion was used to fine-tune the estimate.

These methods have been used for many years and seem to be well applied in this study, says Jonathan Samet, director of the Institute for Global Health at the University of Southern California. But he says the mitigation efforts needed to reduce these impacts continue to struggle. “We don’t have a strategy in place to deal with the many chemicals coming along that our society appears to want and need,” he says, pointing to engineered nanoparticles as one example.

Nsedu Obot Witherspoon, executive director of the U.S.-based Children’s Environmental Health Network, says the new findings provide some additional insight on the global problem, but that even with the gaps and limitations in the study, there already is enough information to support taking more aggressive action on many toxics. “We’re wasting good time,” she says. “We need to jumpstart this quickly. People are dying.”

For example, Witherspoon says she’s surprised some parents still don’t understand the connection between SHS exposure and related health problems in their children. “I know that most parents don’t deliberately want to harm their children,” she says. “So there is still a basic need for more public education messages on the impacts of secondhand smoke.”

On other issues, she says that, absent federal regulation, other jurisdictions can use known remedies that are already proving successful, such as siting new schools and childcare facilities away from high-traffic roads, or eliminating or reducing bus idling near these facilities—strategies that may reduce schoolchildren’s exposures to various toxics.^4^ Another viable strategy, she says, is providing clearer labeling on products so consumers can make better-informed decisions about the chemicals they are exposed to. She also encourages substantially expanding existing biomonitoring programs to cover more people and more toxic substances. Without this, she says, “we are missing a huge piece” of understanding who’s exposed to what chemicals and what these exposures mean for human health.

Witherspoon also suggests that policy makers would benefit from reviewing the underlying assumptions of laws passed long ago that may no longer be on target, such as cigarette ignitability standards for mattresses and mattress pads^5^ in an era when far fewer people are smoking in bed.^6^ And for future efforts conducted by organizations such as the WHO, one of her highest priorities is to track global data on the impacts of consumer product ingredients for which a growing body of evidence suggests adverse health effects, such as bisphenol A, phthalates, and polybrominated diphenyl ether flame retardants.

Expanding beyond the realm of chemical impacts, the WHO and more than 100 experts from around the globe have already evaluated a wide range of environmental factors thought to have a link with 85 significant diseases.^3^ Their definition of the environment is broad and includes all physical, chemical, and biological factors external to individuals, and all related behaviors, but excluding any natural factors that can’t be alleviated in the short or long term with current methods.^3^ Among the specific factors addressed to some degree so far are water, sanitation, and hygiene problems, malnutrition, overcrowding, numerous microbial diseases, and various types of accidents and injuries.

They are concluding that 23% each of all preventable global deaths and DALYs are caused by one environmental factor or another, with large variations among countries in magnitude, sources, and allocation of impacts. The burden ranges from 13% in countries such as Canada, Cyprus, Israel, Singapore, Switzerland, and the United States, to more than one-third in harder-hit countries such as Niger (37%), Angola (36%), Sierra Leone (35%), Burkina Faso (34%), and Mali (33%).

As part of their work, the researchers have developed a fact sheet for each country, in which certain details are broken out. For the latest work on chemicals, only indoor solid fuel use and the burden imposed by outdoor air pollutants, using PM_10_ as a surrogate, are listed. Prüss-Ustün says the sheets provide some initial context, but that country-specific detail on many factors has deliberately been left out. “[We would] rather have the countries estimate these themselves, as they may have more precise estimates of exposure than we do,” she says.

As an example of the information provided on the sheets, the annual death toll in the United States from PM in outdoor air is estimated at 40,600 for the year 2004.^7^ This is a conservative number compared with the U.S. Environmental Protection Agency’s (EPA) estimate of 63,000–88,000,^8^ but it is 69% higher than what was estimated in a study published in 2004 by researchers from the U.S. Centers for Disease Control and Prevention (CDC).^9^ The CDC researchers added another 31,000 deaths from various toxics to determine a total toxics death toll of at least 55,000 in 2000.^10^ Adding the same 31,000 to the top end of the EPA estimate, which does not include deaths from hundreds of outdoor air pollutants, leads to a total of 119,000 deaths. Those assumptions would mean that toxics were the fifth leading killer in 2004, worse than all accidents (112,012 deaths, including 48,053 from transportation-related accidents), all microbial vectors except for influenza and pneumonia (65,275 deaths), influenza/pneumonia (59,664), drug overdoses and adverse reactions (30,711), firearms (29,569), and alcohol (21,081). 11

The numbers indicate toxics are a leading cause of death, but Samet says it’s impossible to determine if these agents are being adequately addressed in the United States. “I have not seen anything like a collective analysis of whether the combination of all regulatory, research, education, and related efforts mesh with the actual death and disease burden for various environmental agents,” he says.

He says the new WHO study could potentially spur greater interest in figuring this out in the United States and other countries, but he wonders if the WHO’s studies and others like them, which have been conducted since the 1990s, are having any real impact. “Do these efforts turn out to be useful to policy makers?” he asks. “It’d be nice to get a better handle on how the world is using them.”

**Leading contributors to premature death and DALYs^1^**

**Indoor solid fuel use**

2.0 mil deaths

41.0 mil DALYs

(75% of burden falls on children under age 15 years)

**Outdoor air pollution**

1.2 mil deaths

8.7 mil DALYs

(10% of DALY burden falls on children under age 15 years)

**Secondhand Smoke**

0.6 mil deaths

10.9 mil DALYs

(61% of DALY burden falls on children under age 15 years)

**Chronic occupational exposures**

0.6 mil deaths

6.8 mil DALYs

**Accidental nonoccupational chemical exposures**

0.2 mil deaths

4.6 mil DALYs

(19% of DALY burden falls on children under age 15 years)

**Suicide attempts with pesticides**

0.2 mil deaths

4.4 mil DALYs

## Figures and Tables

**Figure:**